# Gleanings

**Published:** 1876-02

**Authors:** 


### GLEANINGS.

Professor H. C. Wood gives a clinical lecture upon opium poisoning in the *Medical Times*. He makes a suggestion for an extemporized stomach pump, which is worth quoting: "Pass the end of a piece of gum tubing, three or four feet in length, into the patient's stomach, pour water into the tube until it and the stomach are full, and then drop the fore-end so as to make a siphon. In this way the stomach may be washed out by alternate filling and emptying." He recommends, in deficient respiration, "urging the patient to breathe by vehement commands shouted in the ear; and it is really astonishing how great an effect this may have. I have seen deep inspiration follow such orders in one apparently unconscious." Dr. Wood denies the asserted antagonism between atropia and opium, but says: "Atropia should be used, not as an antidote, but as a remedy when the respiration is failing, precisely as alcohol is used when the circulation is failing." When the respirations go down to four or five in the minute, he administers atropia—one thirtieth of a grain hypodermically. If the breathing progressively increases, he is content, if not, he exhibits one-sixtieth grain every half hour until the effect is produced, or until the dilatation of the pupil warns us to desist. "The golden rule is, give the least possible quantity that will produce the desired effect." He never saw any good effects from the employment of coffee as an antidote, but thinks there may be benefit from cofferia as a cerebal stimulant, especially as an adjunct to the phrenic stimulant, atropia. He denies the value of galvanism, (except in the form of the "wire brush"), as an aid to re-establish the breathing, but commands especially the resort to artificial respiration when the natural functions fail.

Dr. Williamson, of Edinburgh, reports very favorably of the hemostatic power of ergot in hœmoptisis. In forty-four out of fifty cases it rapidly arrested all bleeding. He gives forty minims of the fluid extract at intervals, varying from thirty minutes to two hours.

Dr. Robert Bell, of Glasgow, commends the use the iodide of silver in hooping cough. He uses the eighth of a grain, thrice daily, in children, and finds, in one hundred cases, a *cure* of the disease in four to six weeks. "I would urge all to give this preparation a fair and lengthy trial in the treatment of this disease, as I am convinced that in it we have a most valuable therapeutic agent."

Dr. Alfred Meadows, in the *Obstetrical Journal* of December, discusses the diagnosis of the curable forms of fibroid tumors of the uterus. He calls attention to pain as an indication of a subperitoneal growth, while hemorrhage points to an interstitial or sub-mucous tumor. He regards these two symptoms as pathognomonic as to the seat of the tumor. "The curability of a fibroid tumor of the uterus will, in the main, depend upon its position *quoad* the uterine walls; for instances, if the growth has originated in the wall of the uterus, immediately under its mucous surface, and has grown into the uterine cavity, either in a sessible or pedunculate form; then we say that it is distinctly of the curable class; if, on the other hand, the growth began near the peritoneal surface, and, as it grew, projected more and more into the abdominal cavity, then we say, though not, perhaps, with equal truth, that it is of the incurable class." Displacement of the cervix in any given direction indicates a growth in the opposite direction. A tumor in the posterior wall displaces the cervix entirely, etc. A tumor entirely within the uterus, being central, does not displace the cervix. A closed os, with a small, perhaps normal-sized cervix, and with no abnormal change of its consistence, are, in his experience, almost fatal indications of incurability. The larger the growth and the less the changes in the os and cervix the more hopeless the case is. A dilated os is *pro tanto* a favorable indication of a curable case, and the converse is almost equally true. The larger the cervix the more favorable the case, and, conversely, a small, healthy cervix defies interference—a large one invites it. A fibroid in the posterior lip of the cervix is vastly more frequent than in the interior. The softer, more fleshy, elastic and tense the cervix, the more likely is the tumor removable. The sound determines the length of the uterine cavity, and also the extent to which a sub-mucous fibroid bulges into the cavity.

Of the treatment by hypodermic ergot, he speaks well: "I cannot say that I have actually seen tumors of this sort entirely disappear, but I have certainly seen them reduced considerably in size, and become practically inert." The most satisfactory results have been in cases of small tumors and those of a soft and semi-œdemateous kind. To hope for success with ergot, there ought to be plenty of hemorrhage, or other discharges, and increased size of the uterus, with elongation of its cavity.

Of the surgical treatment to get on to the tumor, if the cervix be thin, small, free from disease and quite uninvaded by the tumor, dilatation is best. On the other hand, if the cervix is large and thick, if the tumor has invaded it, then the cervix should be divided, as tents are of little avail and endanger inflammation. When once we have reached the tumor, the capsule should be broken down with the finger. As a general rule, in cases of this sort, when the capsule is pierced, the detachment of the tumor is very easy, the whole growth may be shelled out from its bed. If this cannot be entirely effected, we may safely trust ergot to accomplish our purpose.

In the after treatment, care must be used against three possible consequences. First, hemorrhage by plugging with cotton, impregnated with carbolic disinfectant, and, if astringent is needed, matico. Secondly, inflammation, by frequent small doses of opium, and constant use of linseed and laudanum poultice, with firm binder over the uterus. Thirdly, septicæmia, by injection of carbolized water, and constant use of vaginal pessary, containing one grain of carbolic acid.

A correspondent of the *British Medical Journal* states that he has under his observation a girl, two years old, who has menstruated regularly for some time. She is in perfect health, and her courses, which are in every respect natural in color, occur every month, causing her much discomfort and pain.

Dr. Kelly, of Philadelphia, reports the *post mortem* delivery of a living child. The mother had died before his arrival, but as the fœtal heart could still be heard, and the os was dilatable, he introduced the hand into the uterus, and delivered by version a mature child, which was successfully revived and afterwards did well. He estimates the time elapsed between the death of mother and delivery of the child at fifteen minutes. It required an hour of faithful work in artificial respiration before the breathing was satisfactorily established.

Br. J. B. Sullivan reports a case of nymphomania cured by excission of the clitorous, after the manner of the late Mr. Baker Brown, of London.

